# PA28 modulates antigen processing and viral replication during coxsackievirus B3 infection

**DOI:** 10.1371/journal.pone.0173259

**Published:** 2017-03-09

**Authors:** Dorota Respondek, Martin Voss, Ina Kühlewindt, Karin Klingel, Elke Krüger, Antje Beling

**Affiliations:** 1 Institut für Biochemie, Charité Universitätsmedizin Berlin, Berlin, Germany; 2 DZHK (German Centre for Cardiovascular Research), partner side Berlin, Berlin, Germany; 3 Institut für Molekulare Pathologie, Universitätsklinikum Tübingen, Tübingen, Germany; University of California, Davis, UNITED STATES

## Abstract

The function of the proteasome is modulated at the level of subunit expression and by association with its regulatory complexes. During coxsackievirus B3 (CVB3) myocarditis, IFN-induced formation of immunoproteasomes (ip) is known to be critical for regulating immune modulating molecules. The function of the IFN-γ-inducible proteasome regulator subunits PA28 α and β, however, in this context was unknown. During viral myocarditis, we found an increased abundance of PA28β subunits in heart tissue. PA28α/β exists in PA28-20S-PA28 and PA700-20S-PA28 hybrid proteasome complexes in cells both with either predominant ip and standard proteasome (sp) expression. Being in line with reduced proteasome activity in PA28α/β-deficient cells, we observed increased levels of oxidized and poly-ubiquitinated proteins upon TLR3-activation in these cells. Moreover, PA28α/β is capable to interfere directly with viral replication of CVB3 and facilitates the generation of CVB3-derived MHC class I epitopes by the proteasome. In contrast to a distinct function of PA28α/β *in vitro*, gene ablation of PA28α/β in mice being on a genetic background with resistance towards the development of severe infection had no significant impact on disease progression. Other than reported for the ip, in this host PA28α/β is dispensable to meet the demand of increased peptide hydrolysis capacity by the proteasome during viral myocarditis.

## Introduction

During infection, replacement of the standard proteasome (sp) by increased formation of the immunoproteasome (ip) with its catalytic subunits LMP2/β1i, MECL-1/β2i and LMP7/β5i represents a cellular mechanism to modulate proteasome activity [1]. In addition to this regulation in target cells of cytokine signalling, the ip is highly abundant in cells of hematopoietic origin including professional antigen presenting cells like dendritic cells [2]. Likewise, a variety of studies have demonstrated that the ip quantitatively modifies proteasomal cleavage preferences and enhances the generation of a significant number of antigenic peptides [3–6]. PA28 is an IFN-γ-induced 11S regulator complex of proteasome cleavage capability that was initially also connected to improved MHC class I antigen processing [7–10]. Two homologous subunits—PA28α (REGα or PSME1) and PA28β (REGβ or PSME2)–form a ring-shaped (180 kDa), heteroheptameric complex by the association of three PA28α and four PA28β subunits [11]. PA28 binds to either one or both ends of the 20S proteasome in an ATP-independent manner [12, 13]. Moreover, it forms a larger complex with PA700 single-capped 20S proteasome complexes resulting in PA700-20S-PA28 particles also referred to as hybrid proteasome [14].

The original observation leading to the identification of PA28 was its capacity to stimulate substantially the peptidase activity of all active sites of the proteasome [12, 13]. Although the exact molecular mechanisms underlying PA28 function are still unclear, several lines of evidence indicate allosteric modification of the 20S proteasome active sites [15], stimulation of peptide entry into the complex [13, 16], stimulation of peptide exit [16, 17] and mediating the binding of the proteasome to components of the endoplasmic reticulum [18]. Crystallographic studies of yeast 20S proteasomes associated with PA26 (the PA28 homologue in *T*. *acidophilum*) demonstrated that binding of PA26 facilitates access to the channel in the proteasome α-ring [17] through which peptide substrates enter into the proteolytic complex [19] and products are released [20]. A quantitative analysis on the rates of protein substrate hydrolysis revealed that PA28-20S particles hydrolyze proteins at identical rates compared to 20S proteasomes [21]. Based on peptide product analysis, Cascio et al. lately proposed a molecular model where PA28 acts as a selective sieve that controls the exit of products from proteasomes based on size and sequence [21, 22]. Such a model is consistent with the biochemical properties of the hybrid proteasome. Degradation assays performed with several denatured proteins showed no effect of PA28 complex formation with PA700-20S proteasome particles on the overall rate of protein degradation. Although the pattern of peptide products was different, the mean size of peptide products was similar between PA700-20S-PA28 and PA700-20S proteasomes [23].

The observation that PA28-containing proteasomes generate a highly divergent pattern of peptide products might be relevant under certain pathophysiological conditions. The increased abundance of PA28-20S ip particles in IFN-γ treated mammalian cells suggests a contribution of this proteasome complex to improved MHC class I antigen processing [21–23]. Althoughexperimental data demonstrated facilitated antigen processing by ip particles *in vivo* [3, 4, 7, 24], mice lacking PA28 complexes had no defects on CD8 T cell responses during infection with Influenza virus [7, 8]. Nevertheless, as shown for the Tyrosinase-related protein (TRP) 2-derived melanoma tumor antigen TRP2_181-188_ [8], PA28 has the capability to modulate the generation of MHC class I epitopes also *in vivo*. Apart from being involved in antigen processing, PA28 has general protective functions under conditions of altered redox homeostasis. The Wang laboratory provided data for an anti-oxidative function of the PA28 complex specifically in cardiomyocytes, all being in line with previous reports by other groups on PA28-20S proteasome complex-dependent proteolysis irrespective of ubiquitin/ubiquitination [25]. PA28 is important for cell fate determination in embryonic stem cells [26] and was connected to reduced obesity-induced ER-stress and insulin resistance in the liver [27].

In our previous studies, we reported on increased formation of ip complexes in heart tissue during viral myocarditis [5, 28, 29]. Substantial *in vitro* evidence was provided for facilitated antigen processing by such ip particles during CVB3 infection [5, 6, 30] and ablation of LMP7 in mice resulted in aggravated myocardial inflammation [29] putatively involving ip-dependent pentraxin3 expression [31]. Nevertheless, the abundance and function of IFN-γ-inducible PA28α/β complexes is still enigmatic during viral myocarditis. In this study, we tackled the question whether and how PA28 might regulate ubiquitin-proteasome function during CVB3 infection. Given that the precise molecular mechanism for the protective function of the ip in cells of the immune system remains to be determined, we also investigated the nature of PA28-20S proteasome and PA28-20S-PA700 hybrid proteasome formation in the context of either standard or immune-20S proteasome core complex expression.

## Materials and methods

### Animals and virus

PA28α/β^-/-^ mice on a C57BL/6 background were provided by Shigeo Murata [8]. 6–8 weeks old male PA28α/β^-/-^ (n = 18) and PA28α/β^+/+^ mice (n = 17) were injected intraperitoneally (i.p.) with 10^5^ PFU CVB3 (cardiotropic Nancy strain) [32]. If not indicated otherwise, mice were anesthetized via isoflurane inhalation and euthanized by cervical dislocation at day 8 after infection. 4 out of 17 CVB3-inoculated PA28α/β^+/+^ mice were not infected as demonstrated by a lack of viral titer in heart tissue. This study was carried out in accordance with the recommendations in the Guide for the Care and Use of Laboratory Animals of the German animal welfare act, which is based on the directive of the European parliament and of the council on the protection of animals used for scientific purposes. The protocol was approved by the Committee on the Ethics of Animal Experiments of Berlin State authorities (G0274/13). All efforts were made to minimize suffering. For the isolation of primary cells, C57BL/6 mice (originally obtained from Jackson Laboratory) and LMP7^-/-^ mice (originally provided by Ulrich Steinhoff) [33] were used. All mice were kept at the animal facilities of the Charité University Medical Center.

### Cell culture, infection and transfection

Primary murine cardiomyocytes (eCMs) were isolated from E14 embryonic mouse hearts obtained from wild-type C57BL/6 and PA28α/β^-/-^ mice as described previously [34]. Mouse bone marrow-derived macrophages (BMMs) were isolated by flushing the femur and tibiae with pre-warmed RPMI 1640 medium (Biochrom) and cultured according to the methods described recently [31]. BMMs were used after approximately eight days of culture. HeLa cells were purchased from ATCC and cultured in Dulbecco’s modified Eagle’s medium containing 10% (v/v) fetal calf serum and 1% (v/v) penicillin/streptomycin. Primary splenocytes were isolated from 8–12 weeks old wild-type and PA28αβ^-/-^ mice. Spleen was passed through a 40-μm cell strainer in ice-cold phosphate-buffered saline (PBS). Cells were pelleted and re-suspended in ACK lysis buffer (150 mM NH_4_Cl, 10 mM KHCO_3_, 0.1 mM Na_2_EDTA). After 2 min incubation at room temperature PBS was added and cells spun down. Splenoyctes were cultured in RPMI (Biochrom) supplemented with fetal calf serum (10%, Biochrom), 50 μM mercaptoethanol (Roth), penicillin/streptomycin (1%, Biochrom), L-glutamin (2 mM, Biochrom). Murine embryonic fibroblasts (MEFs) were isolated from E14 embryos obtained from wild-type C57BL/6 and PA28α/β^-/-^ mice. In brief, embryos were minced in 0.05% trypsin and incubated at 37°C for 5 min. Cells were detached by pipetting up and down. After incubating cells at 37°C for further 10 min, medium was added to stop trypsin reaction and cells were passed through a 100-μm cell strainer and pelleted by centrifugation. MEFs were cultured in Dulbecco’s modified Eagle’s medium with low glucose (Biochrom) containing fetal calf serum (10%, Biochrom) and L-glutamin (2 mM, Biochrom).

CVB3 infection was conducted in eCM and HeLa cells at a multiplicity of infection (MOI) of 0.01 or 0.1 as recently described [31]. For all *in vitro* assays on cell viability, BMMs were stimulated with polyinosinic:polycytidylic (poly-I:C) acid (50 μg/ml, Invivogen). For inhibition of PA28α/β complex expression, HeLa cells were seeded into 12-well plates at a density of 5x10^4^ cells per well. 24 h post seeding cells were transfected with (i) PA28α and PA28β siRNA (each 30nM, ON-TARGET plus—SMART pool, Dharmacon) or with (ii) control siRNA (60 nM, ON-TARGET plus—SMART pool, Dharmacon) using X-tremeGENE siRNA transfection reagent (Roche) and cultured for 72 hours. For overexpression studies, HeLa cells were seeded into 12-well plates at a density of 1.5 x 10^5^ cells per well and transfected the following day with (i) hPA28α-pcDNA3.1 and hPA28β-pcDNA3.1 (0.25 μg each/well) [35] or (ii) mPA28α-pSG5 and mPA28β-pSG5 (0.25 μg each/well) for 24 hours using polyethylenimine (Polysciences). Empty pcDNA3.1 and pSG5 served as a control, respectively.

### Histology

Histological staining of murine heart tissue sections was performed as described elsewhere [36].

### Determination of viral titers

Plaque assays were performed on sub-confluent green monkey kidney cells as described recently [36].

### RNA isolation and quantitative real-time PCR

RNA preparation and cDNA synthesis were performed as described recently [36]. Quantitative real-time PCR was performed using following primers and TaqMan^®^ probes: CVB3—fw: 5’-TCCTCCGGCCCCTGA-3’, rev: 5’-GATTGTCACCATAAGCAGCCA-3’, probe: 5’-FAM-CGGAACCGACTACTTTGGGTGTCCGT-TAMRA-3’; murine PA28α (PSME1 Mm00650858_g1, Life Technologies), murine PA28β (PSME2, Mm01702833_g1, Life Technologies); murine HPRT—fw: 5´-ATCATTATGCCGAGGATTTGGAA-3´, rev: 5´-TTGAGCACACAGAGGGCCA-3’, probe: 5’FAM-TGGACAGGACTGAAAGACTTGCTC GAGATG-TAMRA-3’; human PA28α (PSME1, Hs00389209_m1, Life Technologies); human PA28β (PSME2, Hs01581609_g1, Life Technologies), human HPRT—fw: 5´-AGTCTGGCTTATATCCAACACTTCG-3´, rev: 5´-GACTTTGCTTTCGGTCAGG-3’, probe: 5’-TTTCACCAGCAAGCTTGCGACCTTGA-3’. The cDNA amplification was measured using StepOnePlus^™^ Real-Time PCR System. mRNA expression was normalized to Hypoxanthin-Guanin-Phosphoribosyl-Transferase (HPRT).

### DCFH-DA staining

To measure cellular reactive oxygen species (ROS), 2',7'-dichlorodihydrofluorescein diacetate (DCFH-DA, Sigma Aldrich) was used according to previously described protocols [34]. In brief, after treatment with poly-(I:C), cells were washed twice with warm Hank's buffered salt solution (HBSS, Gibco) and incubated with DCFH-DA (10 μM) dissolved in the cell culture medium without any supplements at 37°C in the dark for 60 min. Intracellular ROS irreversibly oxidize DCFH-DA to fluorescent 2',7'-dichlorofluorescin (DCF). Afterwards, cells were washed twice with HBSS to remove remaining DCFH-DA and 200 μl of FACS buffer (0.5% (v/v) BSA and 0.01% (v/v) NaN_3_ in PBS) were added and cells were collected using cell scraper. The DCF fluorescence was captured by a FACSCalibur cytometer (BD Bioscience) and analysed using FlowJo7 software.

### Cell viability assay

Cellular metabolic activity was assessed using MTT-assay. Therefore, BMMs cultures were seeded in 96-well-plates (flat bottom, Greiner). Following poly-(I:C) treatment, cells were washed with PBS and incubated with 500 μg/ml 3-(4,5-dimethylthiazol-2-yl)-2,5-diphenyltetrazolium bromide (MTT, Sigma Aldrich,) in complete culture medium at 37°C for 2 hours. Resulting formazan crystals were solubilized in isopropanol/acetic acid (95/5, v/v). The absorbance was measured at 570 nm (Bio-Tek Synergy HT). Induction of programmed cell death was analysed with the fluorescence based Apo-ONE^®^ Homogeneous Caspase-3/7 Assay (Promega) according to manufacturer’s instructions.

### Proteasome and PA28 purification

20S proteasomes were purified from human lymphoblast T2 cells (standard proteasome) and T2.27 cells (immunoproteasome). Cells were homogenized in a five-fold volume (v/w) of TEAD buffer (20 mM Tris-HCl, 1 mM EDTA, 1 mM NaN_3_, 1 mM DTT, pH 7.0) by douncing (20 strokes at 4°C). Following centrifugation at 15,000 rcf at 4°C for 40 min, the supernatant was used for purification of 20S proteasomes as described elsewhere [37]. PA28 was isolated from human erythrocytes as previously described [38].

### Protein isolation and immunoblot analysis

For SDS-PAGE cell or tissue lysis was performed using RIPA buffer (20 mM Tris-HCl pH 7.5, 100 mM NaCl, 10 mM EDTA, 1% (v/v) Nonidet P40, 0.1% (w/v) SDS, 10 μM MG132, 5 mM NEM, Complete^®^ protease inhibitor cocktail (Roche)).

For native-PAGE, cells were lysed with TSDG buffer (10 mM Tris-HCl pH 7.0, 25 mM KCl, 10 mM NaCl, 1.1 mM MgCl_2_, 1 mM DTT, 2 mM ATP, 10% (v/v) glycerol) followed by 4 cycles of snap freezing and thawing.

Immunoblot analysis was performed according to standard procedures [34]. Primary antibodies: murine PA28α (K232/2, lab stock), murine PA28β (K231/2, lab stock), human PA28α (Cell Signaling), human PA28β (Cell Signaling), α4 (K378/1, lab stock), LMP7 (K63, lab stock), Rpt6 (Enzo Lifesciences), VP1 (DAKO), Ubiquitin (DAKO), DNP (Sigma), actin (Millipore), human GAPDH (Santa Cruz), mouse GAPGH (GeneTex). The bound primary antibodies were detected using either (i) IRDye800CW labelled goat anti-mouse/anti-rabbit secondary antibodies in conjunction with an Odyssey CLx infrared imaging system (Li-Cor Biosciences) or (ii) horseradish peroxidase (HRP)-labelled goat anti-mouse/anti-rabbit secondary antibodies (Santa Cruz) using an enhanced chemiluminescence light (ECL) detection kit (GE Healthcare) and a PEQLAB Fusion FX Imaging System.

### Oxyblot

Detection of carbonyl groups introduced into proteins by oxidative modification was performed as follows: cellular extracts generated in RIPA buffer (5 μg protein in total volume of 5 μl, without NEM) were mixed 1:1 with 12% (w/v) SDS and subsequently incubated with 2,4-dinitrophenylhydrazin (DNP, 5 mM) in the dark for 30 min. DNP-derivatization was stopped using 7.5 μl of neutralizing buffer (2 M Tris in 30% (v/v) glycerol). After addition of DTT (3 μl of 10 mM stock) protein samples were separated by 8% SDS-PAGE.

### Activity-based probe assay

Accessibility to active sites of the proteasome complexes was investigated with activity-based probes (ABP). Cellular extracts in TSDG buffer were incubated with pan-reactive ABP BodipyTMR-Ahx3L3VS [39] (MV151, 2 μM) at room temperature in the dark for 30 min. Following native-PAGE, fluorescence signals were detected using a VersaDoc MP 4000 (Bio-Rad). For the measurement of proteasome activity in splenocytes, 5×10^5^ cells were incubated with pan-reactive activity-based probe BodipyFL-Ahx3L3VS [40] (500 nM) at 37°C in 95% humidified atmosphere and 5% CO_2_ for 2 hours. Subsequently, cells were washed twice with PBS containing 1% BSA and 2 mM EDTA. Fluorescence of ABP was detected by flow cytometry (FL-1) on a FACSCalibur cytometer (BD Bioscience) and analysed using FlowJo7 software.

### Analysis of MHC class-I surface expression on mouse embryonic fibroblasts

MHC class I regulation after IFN-β stimulation was examined in MEFs using anti-mouse MHC I (H-2Kb, AF6-88.5.5.3, eBioscience) and corresponding isotype control IgG2aκ (eBM2a, eBioscience). 1–2×10^5^ cells were stained in PBS containing 1% BSA at 4°C in the dark for 20 min. Subsequently cells were washed twice with PBS containing 1% BSA and 2 mM EDTA and analysed by flow cytometry.

### Proteasome separations by glycerol gradients centrifugation

Homogenous 20%– 40% glycerol gradients were prepared in Ultra Clear^™^ centrifugation tubes (Beckman Coulter) from 20% and 40% glycerol solutions (10 mM Tris-HCl pH 7.0, 25 mM KCl, 10 mM NaCl, 1 mM DTT, 1.1 mM, MgCl_2,_ 2 mM ATP) using a Gradient Master IP 107 Mixer. BMMs lysates were prepared in TSDG buffer as described above. 4 mg of protein in a maximal volume of 500 μl were loaded onto the gradients and subjected to ultra-centrifugation with SW40 rotor at 280,000 rcf (4°C, Beckman Coulter) for 20 hours. Subsequently, 500-μl fractions were collected from the top of the gradient. Chymotryptic-like activity was assessed in each fraction measuring the hydrolysis of the fluorogenic peptide Suc-LLVY-AMC (Bachem). 10 μl of each fraction was incubated in a black 96-well plate (Greiner) with 90 μl of 100 μM Suc-LLVY-AMC in assay buffer (50 mM Tris-HCl pH 7.2, 1 mM DTT, 0.5 mM EDTA) in the dark at 37°C for 30 min. The released AMC was detected was monitored fluorimetrically at Ex/Em = 360/460 nm (Bio-Tek Synergy HT). Aliquots of the proteasome-containing glycerol gradient fractions (300 μl) were ethanol-precipitated, resuspended in Laemmli buffer, applied onto 15% SDS-polyacrylamide gels and subjected to Western blotting of proteasomal proteins. Remaining aliquots (180 μl) of fractions containing high-molecular proteasome complexes (fractions 6–10: 31%– 35% glycerol) were concentrated to a volume of 30 μl using Amicon^®^ Ultra centrifugal filters (Millipore). 24 μl of each fraction was mixed with 8 μl of 4-fold sample buffer (Life Technologies) and subjected to native-PAGE for detection of PA28β by immuno-blotting.

### *In vitro* fragmentation of synthetic polypeptides and mass spectrometry

Synthetic peptides harbouring CVB3 MHC class I epitopes P3D_2170-2177_ (P3D_2158-2185_: RKIRSVPVGRCLTLPAFSTLRRKWLDSF) [5] and VP2_272-302_ (VP2_285-293_: VMPYTNSVPMDNMFRHNNVTLMVIPFVPLDY) [5, 6] were obtained from the in-house peptide synthesis core facility (Institute for Biochemistry, Charité Universitätsmedizin Berlin). Peptides (40 μM) were incubated with 20S proteasome and PA28 in 100 μl of TEAD buffer at 37°C for indicated periods. The ratio of 20S proteasome and PA28 was 2.5 μg 20S proteasome / 0.6 μg PA28 for CVB3 P3D_2158-2185_ and 5 μg 20S proteasome / 1.2 μg PA28 for CVB3 VP2_272-302_. Reactions were terminated by adding trifluoroacetic acid (TFA) to a final concentration of 0.3%. Samples were analyzed by RP-HPLC. Peptide separation was performed with a sample volume of 10 μl on a 1-mm reverse phase column (Beta Basic-18, 100-mm×1-mm, 3 μm, 150 Å) using a Surveyor system (both Thermo Fisher Scientific, U.S.A.). The mobile phase (A) was 0.05% (v/v) TFA in water and (B) was 70:30 (v/v) acetonitrile/water containing 0.045% (v/v) TFA. For peptide identification, online mass spectrometry (ms) analysis was carried out on a DECA XP MAX iontrap instrument (Thermo Fisher Scientific, U.S.A.). MS data were acquired with a triple scan method in positive ion mode (MS-mass range 250–2000 *m/z*, zoom scan, MS/MS). Analysis of ESI/MS data was accomplished using Bioworks 3.3 (Thermo Fisher Scientific, U.S.A.). Database search was performed using a fasta databases of the peptides and the following parameters: no enzyme, mass tolerance for fragment ions 1 amu. To monitor epitope generation at the indicated points in time, identified peaks were followed by LCQuan software version 2.5 (Thermo Fisher Scientific, U.S.A.).

### Statistics

Results are expressed as mean ±/+ standard error of mean (SEM). Logarithmic data (semi-quantitative RNA quantification) measured on a linear scale was transformed logarithmically prior to data plotting and data analysis. For two group comparisons of normally distributed parameters, unpaired t-test was applied. In case of detection of unequal variances (F-test), unpaired t-test with Welch’s correction was applied. For two group comparisons of not normally distributed parameters, Mann-Whitney test was performed. For multiple group comparison, unequal variance versions of one- or two-way ANOVA were performed followed by a Tukey-Kramer’s multiple comparison procedure. The significance threshold was set to 0.05. Software for data analysis: GraphPad Prism version 6.00 (GraphPad Software, La Jolla California USA).

## Results

### Regulation of PA28α/β protein levels during CVB3 infection

**Fig 1:**
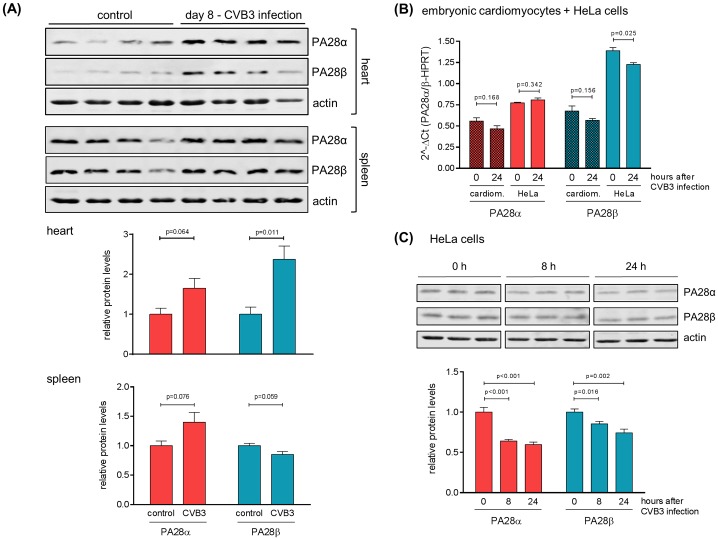
Regulation of PA28αβ during CVB3 infection. (A) 6–8 weeks old, male C57BL/6 mice (n = 4) were infected with CVB3 and sacrificed at day 8 after infection. Age- and gender-matched naive mice (n = 4) served as a control. Tissue homogenates of heart and spleen were analyzed by Western blotting to determine PA28α and PA28β protein levels. Actin served as a loading control. Densitometric analysis was performed and PA28α/PA28β levels are shown as relative intensities with a normalization based on the respective levels of actin (means + SEM, n = 4). (B) Primary embryonic cardiomyocytes (eCMs) obtained from wild-type mice and HeLa cells were infected with CVB3 at MOI 0.1 for 0 or 24 h. PA28α/PA28β mRNA levels were determined by real-time qPCR (means + SEM, n = 3). (C) HeLa cells were infected with CVB3 at MOI 0.1 for indicated lengths of time. PA28α and PA28β protein levels in total protein extracts were determined by Western blotting. The densitometric analysis of PA28α/PA28β protein levels is depicted as relative intensities (means + SEM, n = 3).

It was proposed that the generation of highly divergent peptide pattern attributed to the increasing abundance of PA28-ip complexes might be relevant under pathological conditions like viral infection. Following the aim to investigate PA28α/β abundance during viral myocarditis, PA28α/β levels were monitored in heart tissue homogenates obtained at day 8 after CVB3 infection, which represents the peak of the inflammatory response in C57BL/6 mice [28]. Murine heart tissue contains baseline amounts of PA28α/β subunits. Consistent with a robust ip formation during viral myocarditis [5, 28, 29], we found an increased abundance of PA28α/β subunits comprising a significant rise in PA28β levels in infected mouse hearts compared to naive mice (Fig 1A). Baseline PA28α/β levels found in spleen of naive mice were not significantly affected during CVB3 infection. Next, we questioned whether target cells of CVB3 infection like cardiomyocytes or HeLa cells directly respond with altered PA28α/β expression upon viral infection. We found stable PA28α and β transcripts in embryonic cardiomyocytes obtained from wild-type mice. Although PA28α transcripts were not significantly affected in CVB3-infected HeLa cells, we observed reduced PA28β mRNA levels. Protein levels both of PA28α as well as PA28β were significantly reduced during CVB3 infection (Fig 1B/1C) indicating a direct interference of viral proteins with PA28α/β protein stability.

### Protein homeostasis in TLR3-activated bone marrow derived macrophages

**Fig 2:**
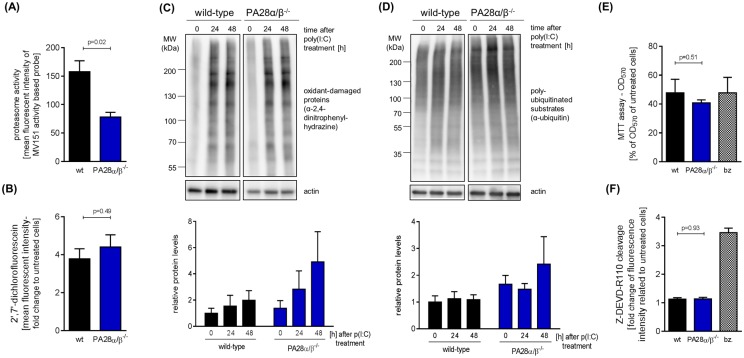
Protein homeostasis in TLR3-activated bone marrow derived macrophages. (A) Splenocytes freshly isolated from 8–12 weeks old wild-type and PA28αβ^−/−^ mice were incubated with MV151 activity based probe (ABP). Binding of ABP was detected by flow cytometry and is shown as mean fluorescence intensity (means + SEM, n = 4 for wild-type, n = 3 for PA28α/β^−/−^, 2 independent experiments). Bone marrow derived macrophages (BMM) were obtained from 8–10 weeks old wild-type and PA28αβ^−/−^ mice. BMMs were treated with poly(I:C). (B) After 24 hours of stimulation, cells were incubated with DCFH-DA to measure reactive oxygen species (ROS). Resulting DCF fluorescence was detected by flow cytometry and mean fluorescence intensity is shown as fold change relative to untreated cells (means + SEM, n = 3). (C) BMM lysates obtained after 0, 24, and 48 hours of treatment with poly(I:C) were subjected to Oxyblot analysis (depicted results were obtained from the same membrane). Results of a densitometric analysis are shown as relative intensities in comparison to untreated wild-type BMM (means + SEM, n = 3, no significant differences were detected). (D) Total cell lysates obtained after 24 and 48 hours of poly(I:C) treatment were subjected to Western blotting of poly-ubiquitinated proteins and actin (depicted results were obtained from the same membrane). Densitometric analysis was performed and levels of poly-ubiquitin conjugates are shown as relative intensities in comparison to untreated, wild-type BMM (means + SEM, n = 3, no significant differences were detected). Actin served as a loading control and was used for normalization. Cellular viability was assessed 24 hours after poly(I:C) treatment by (E) MTT assay and by (F) Apo-ONE assay presented as fold change relative to untreated wild-type cells (means + SEM, n = 3). In both assays bortezomib (bz) treatment served as a positive control.

FACS analysis of splenocytes incubated with the pan-reactive activity based probe MV151 revealed that gene ablation of PA28α/β results in a significant decrease of cellular proteasome activity (Fig 2A). As the abundance of PA28α/β levels is directly affected by CVB3 infection, we decided to circumvent viral infection experiments and address PA28 function by using polyinosinic:polycytidylic acid (polyI:C) that mimics double-stranded viral RNA replication intermediates and binds to TLR3 receptors. After day 8 of CVB3 infection, both inflammatory monocytes and macrophages represent the most frequently detected types of immune cells in heart tissue [unpublished observation]. To address the question whether in these cells PA28 is involved in controlling redox and protein homeostasis during infection, we examined polyI:C-treated bone marrow derived macrophages (BMM) 24 and 48 hours after TLR3-stimulation. This treatment resulted in the formation of reactive oxygen species (ROS) (Fig 2B) and consequently in protein oxidation that was slightly more induced in BMMs derived from PA28α/β^-/-^ mice in comparison to cells from wild-type mice (Fig 2C). In line with this observation, PA28α/β deficiency in BMMs resulted in a slight increase of the overall level of poly-ubiquitination (Fig 2D). However, gene ablation of PA28α/β had no aggravating effect on polyI:C-induced cytotoxicity (Fig 2E/2F).

### Effect of PA28α/β on coxsackieviral replication

**Fig 3:**
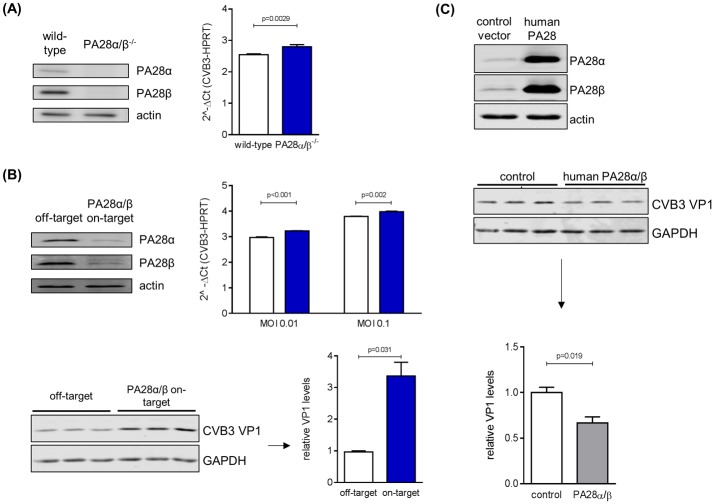
Effect of PA28α/β on coxsackieviral replication. (A) eCMs were isolated from wild-type as well as PA28α/β^−/−^ mice and cell lysates were subjected to Western blotting of PA28α and PA28β (top). Cells were infected with CVB3 at MOI 0.1 for 16 hours and CVB3 mRNA was determined by real-time qPCR (bottom). Data are means + SEM of n = 4 independent experiments. (B) HeLa cells were transfected with siRNA targeting PA28α/β for 72 hours and protein levels were assessed by Western blotting of PA28α and PA28β (top left). siRNA-treated HeLa cells were infected with CVB3 at indicated MOIs for 16 hours and relative expression of CVB3 mRNA was determined by real-time qPCR (bottom, left graph, means + SEM of n = 2). In addition, lysates of CVB3-infected HeLa cells (MOI 0.01) pre-treated with siRNA were analyzed by Western blotting of CVB3 VP1 structural protein and GAPDH (top right). Densitometric analysis of VP1 levels, normalized to GAPDH, is depicted as relative intensities in comparison to infected HeLa cells pre-treated with off-target siRNA (means + SEM, n = 3). (C) HeLa cells were transiently transfected with human PA28α/β. Cell lysates were subjected to Western blotting to confirm recombinant PA28α/β expression. Following 24 hours PA28α/β transfection, cells were infected with CVB3 at MOI 0.1 for 16 hours. VP1 and GAPDH levels were determined by Western blotting and quantified by densitometry of the immune blots.

There is experimental evidence that human immunodeficiency virus-1 Tat protein [41] and hepatitis B virus HBx peptide 116–138 [42] compete with PA28 for binding to 20S core particles. As the coxsackieviral replication machinery at least partially relies on intact function of the ubiquitin-proteasome system (UPS) [43, 44], we investigated whether PA28 is capable to affect CVB3 replication. Therefore, viral RNA genome copy numbers and the expression of the CVB3 structural protein VP1 were examined with regard to PA28α/β abundance in primary embryonic cardiomyocytes (eCM). Infection of eCM obtained from PA28α/β^-/-^ mice resulted in a significant increase of viral genome copy number (Fig 3A). Next, we took advantage of CVB3-permissive HeLa cells. Consistent with our finding in eCM, reduction of PA28α/β expression by siRNA treatment resulted in a significant increase of CVB3 genomes and VP1 protein levels (Fig 3B). Although PA28α/β levels have an impact on viral genome and protein synthesis, the amount of released viral particles was not altered by PA28α/β siRNA treatment (data not shown). In another experimental set-up, we aimed to effectuate an increase in PA28 expression in HeLa cells. As IFN-γ stimulation results in potent suppression of CVB3 replication [unpublished observation], we made use of PA28α/β cDNA constructs resulting in significant PA28α/β overexpression. Infection of PA28α/β-overexpressing HeLa cells resulted in reduced VP1 protein levels 16 hours after CVB3 infection (Fig 3C). Altogether, these *in cellulo* findings are indicative for a putative role of PA28α/β during CVB3 replication.

### Influence of PA28α/β on proteasomal processing of CVB3-derived MHC class I peptides

**Fig 4:**
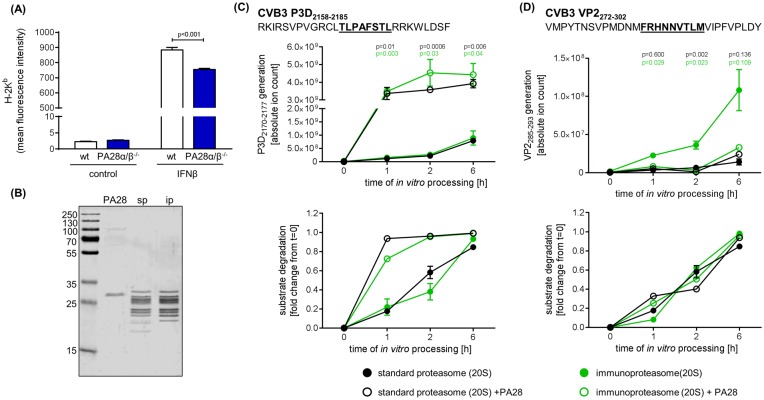
Influence of PA28α/β on MHC class I antigen processing of CVB3 epitopes. (A) Murine embryonic fibroblasts were treated with IFN-β for 24 hours. For assessing MHC class I expression, cells were stained with anti-H-2K^b^ mAb and analyzed by flow cytometry (mean + SEM, n = 3). (B) For *in vitro* peptide processing studies PA28 from human erythrocytes, 20S sp from human lymphoblasts (T2 cells) and 20S ip from stably LMP2/LMP7-transfected T2 cells (T27 cells) were purified. The purity of the preparations was examined by Coomassie staining of a SDS-PAGE gel. Two epitope-harboring CVB3 peptides—the 28-mer polypeptide P3D_2158-2185_ (C) and the 31-mer polypeptide VP2_272-302_ (D)–were processed in the presence of T2-derived sp (filled black circles) and T27-derived ip (filled green circles) for the indicated incubation periods. Open circles represent results of proteasome fragmentation studies in the presence of PA28 for sp (black) and ip (green). The graphs in the upper panel summarize the generation of the respective CVB3 epitopes (C) P3D_2170-2177_ and (D) VP2_285-293_ detected by ESI–MS/MS. The lower graphs depict the substrate degradation as fold changes of the ion counts at point in time = zero. All ion counts of peptide substrates were normalized to the amount of the respective substrate detected in the sp assay at point in time = zero. All data are means ± SEM of two technical replicates. Unpaired t-tests were performed at each point in time to compare ion counts for P3D_2170-2177_ and VP2_285-293_ in the absence (filled circles) or presence of PA28 (open circles). All experiments shown are representative for at least two independent experiments.

PA28 was originally found as an activator of 20S proteasome influencing the hydrolysis of short fluorogenic peptides [12] and later linked to facilitated antigen processing [10]. Taking into account that CVB3 potently suppresses MHC class I antigen presentation [45] and only immunosubdominant epitopes eliciting weak CD8 T cell responses have been characterized [5, 6, 46], investigation of PA28 function for CVB3 antigen processing is restricted to *in vitro* peptide processing studies. First, we analysed the amount of overall MHC class I expression on the cell surface of murine embryonic fibroblasts (MEF) that were stimulated with IFN-β. Such treatment resulted in robust up-regulation of H2-K^b^ expression on MEFs that were isolated from wild-type mice. Ablation of PA28α/β coincided with a significant reduction of IFN-β-induced H2-K^b^ expression in MEFs (Fig 4A). For *in vitro* antigen processing studies, we took advantage of two previously characterized peptide substrates—the VP2_272-302_ and P3D_2158-2185_ harbouring H2-D^b^-restricted CVB3 VP2_285-293_ [5, 6] and H2-K^b^-restricted CVB3 P3D_2170-2177_ epitopes [5], respectively. These peptide substrates were processed *in vitro* by sp and ip isolated from T2 and T27.7 cells in the presence of PA28α/β complexes (Fig 4B). PA28α/β resulted in a profoundly enhanced generation of the P3D_2170-2177_ epitope by both sp and ip, without an additional effect of the nature of the 20S proteasome complex studied (Fig 4C). Facilitated antigen processing coincided with increased turnover of the P3D_2158-2185_ peptide substrate. For the VP2_272-302_ peptide substrate, we confirmed previous observations of improved VP2_285-293_ liberation by the ip in comparison to the sp [6]. Whereas PA28 had no impact on VP2_285-293_ generation by the sp, we found reduced amounts of ip-dependent VP2_285-293_ liberation in the presence of PA28 (Fig 4D).

### Characterization of PA28-proteasome complexes in wild-type and LMP7^-/-^ macrophages

**Fig 5:**
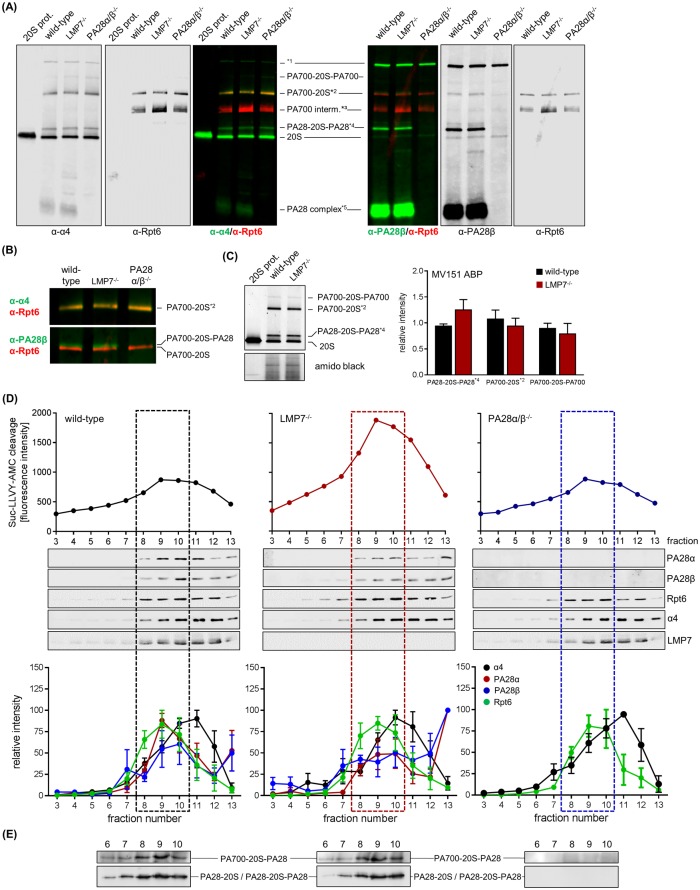
Characterization of PA28-proteasome complexes in wild-type and LMP7^−/−^ macrophages. (A) Cellular extracts were generated from wild-type, LMP7^−/−^ and PA28α/β^−/−^ BMMs. Proteins were separated by native PAGE and subjected to Western blotting of proteasome core (α-α4) and PA28 complex (α-PA28β) co-stained for PA700 complex (α-Rpt6), respectively. 20S proteasome purified from mouse B8 fibroblasts was applied for comparison. Fluorescence dye-coupled secondary antibodies were used for detection. *1 –unspecific signal, *2 –PA700-20S and PA700-20S-PA28, *3 –PA700 intermediate complex [51], 4*–PA200-20S-PA200 [52], 5*–PA28α/β not bound to a proteasome core. (B) Magnified section of the α-α4/α-Rpt6 and α-PA28β/α-Rpt6 double-staining from (A) depicting the high molecular mass proteasome complexes PA700-20S and PA700-20S-PA28. (C) Expression and accessibility of catalytically active proteasome subunits within different proteasome-proteasome regulator complexes was investigated with MV151 pan-reactive ABP binding to all active sites and analyzed by densitometry (means + SEM of n = 3). Amido black staining indicates equal protein loading. (D) Total BMM protein extracts were subjected to glycerol density gradient centrifugation to separate distinct proteasome complexes. Proteasome-dependent peptide hydrolysis was determined in the obtained fractions by cleavage of the short fluorogenic peptide Suc-LLVY-AMC. Glycerol gradient fractions 3–13 (29%– 37.5% glycerol) containing high and low molecular mass proteasome complexes were concentrated and subjected to SDS-PAGE and Western blot analysis of proteasome subunits. Densitometric analysis of immune-blots is represented as relative intensities normalized to the most intense signal, respectively (means ± SEM, n = 4). Dotted boxes depict glycerol gradient fractions with the highest abundance of PA700-20S-PA28 complexes as determined by native PAGE (E) and immuno-blotting of PA28β (D).

IFN-γ stimulation triggers the concomitant up-regulation of the catalytic subunits of ip and PA28α/β complexes resulting in formation of PA28-ip particles. Likewise, previous biochemical studies used PA28-i20S-PA28 and PA700-i20S-PA28 complexes in comparison to non-PA28-capped i20S or PA700-i20S particles for characterizing proteasome function in the presence of bound PA28α/β complexes [21, 23]. As depicted in Fig 1, we found high abundant PA28 expression in spleen with no significant alteration during CVB3 infection. TLR3 stimulation of BMM, which show considerable expression of ip and sp as well as PA28α/β under latent conditions, had no additional effect on PA28α/β levels either [unpublished data]. Therefore, in such resting immune cells the function of the ip, but also the sp might be affected by PA28 complex formation. In fact, taking into account data on the structural resolution of proteasome and PA28α/β association [17, 19, 47, 48], any influence of the composition of the catalytic subunits within the 20S core particle on proteasome complex formation with PA28 appears to be unlikely. Nevertheless, considering that in presence of PA28 we found differential effects on proteasomal epitope liberation, we investigated the general capability of PA28 complexes to interact both with sp and ip complexes. BMM obtained from wild-type mice served as a model for predominant ip expression and BMM from LMP7^-/-^ mice were used for high abundant sp expression [49]. Investigation of proteasome and PA28 complex association as conducted by native-PAGE Western blot analysis (Fig 5A/5B) and activity-based probe profiling (Fig 5C) revealed a similar abundance of PA28-20S-PA28 and PA700-20S-PA28 complexes in both homogenates obtained from wild-type and LMP7^-/-^ BMM. Moreover, the absolute abundance of 20S and PA700-20S complexes was similar in both cell types indicating that the nature of the catalytic subunits most likely neither influences the absolute abundance of the proteasome core complex nor proteasome complex formation with its regulator PA28 and PA700. In an alternative approach, we examined proteasome modulator binding to 20S proteasome complexes upon fractionating proteins in cellular homogenates using glycerol gradient centrifugation. As illustrated in Fig 5D, the peak Suc-LLVY-AMC cleavage that indicates maximal chymotryptic-like proteasome activity was found in fractions 8–10 (31%– 33% glycerol) in lysates obtained from wild-type and LMP7^-/-^ BMMs, respectively. Moreover, we found a similar abundance of PA700-20S-PA28 and PA28-20S / PA28-20S-PA28 particles for the respective 20S core types of ip and sp (Fig 5D/5E). Relative quantification of PA28α and PA28β as well as α4 (20S core particles) and Rpt6 (PA700 regulator) revealed no differences between BMM with ip (wild-type) and sp (LMP7^-/-^) expression (Fig 5D). Consistent with our previous findings [28] and reports of other groups [49, 50], we observed increased Suc-LLVY-AMC cleavage in cellular homogenates obtained from wild-type BMM with abundant ip amounts in comparison to extracts obtained from LMP7^-/-^ BMM with predominant sp expression (Fig 5D). Interestingly, the ablation of PA28α/β had only a minor impact on the chymotryptic-like proteasome activity as evidenced by slightly altered amounts of AMC release from the respective short fluorogenic peptide substrate in comparison to wild-type mice (Fig 5D). In summary, using BMM isolated from wild-type and LMP7^-/-^ mice as a model for predominant ip and sp expression, we assume that under native conditions both ip and sp 20S proteasomes interact with its regulators PA700 and PA28 with similar efficacy.

### Investigation of PA28α/β^-/-^ mice during CVB3 myocarditis

**Fig 6:**
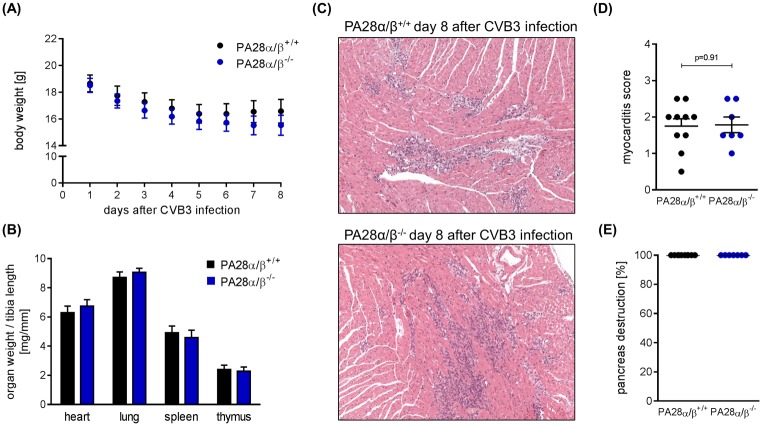
Investigation of PA28αβ^-/-^ mice during CVB3 myocarditis. PA28^+/+^ and PA28^-/-^ mice were infected with CVB3. (A) Body weight was monitored daily and mice were sacrificed at day 8 post infection (means ± SEM). (B) Heart, lung, spleen and thymus weight was determined and normalized to the respective tibia length (means + SEM). (C) Representative haematoxylin/eosin (HE) staining of cardiac tissue sections obtained from CVB3-infected PA28^+/+^ (n = 10) and PA28^−/−^ (n = 7) mice are shown. (D) Myocardial damage comprising cardiac cell necrosis, inflammation, and scarring was quantified applying a myocarditis score from 0 to 4 [36]. (E) The severity of pancreas tissue destruction upon CVB3 infection was rated on a scale from 0% to 100% on HE stains of pancreatic tissue sections.

Our observation that PA28α/β affects both antigen processing by the proteasome and coxsackieviral replication is indicative for a putative function of this proteasome activator during CVB3 myocarditis. To investigate such proposed biological effects *in vivo*, we made use of mice lacking both PA28α and PA28β expression but have intact ip formation [8]. PA28α/β^-/-^ mice used in this study were on a C57BL/6 background, which is connected to low susceptibility to viral myocarditis [32]. Infection of PA28α/β^-/-^ mice and respective littermates revealed a redundant role of PA28α/β *in vivo*. General signs of viral infection such as weight loss were not different in both PA28α/β^-/-^ and PA28α/β^+/+^ mice (Fig 6A). We found no difference in the myocardial lesions reflecting both immune cell infiltration and necrosis of cardiomyocytes as a result of direct cytolysis by CVB3 infection (Fig 6B–6D). Consistent with this similar pattern of myocardial injury in both hosts, we detected no difference in CVB3 titers in heart tissue at day 8 after infection (data not shown). Altogether, this *in vivo* study indicates that genetic ablation of PA28α/β can be efficiently compensated and argues against a significant biological impact of PA28α/β on both MHC class I antigen presentation and regulation of viral replication during CVB3 infection in C57BL/6 mice.

## Discussion

The molecular mechanisms and cellular consequences of PA28α/β function are poorly understood. In the present study, using *in vitro* models of CVB3 infection and proteasomal antigen processing as well as an *in vivo* model of CVB3 myocarditis, we investigated the biological function PA28α/β complexes during virus-induced heart muscle inflammation. As PA28α/β complexes can associate with sp and ip 20S core particles resulting in the formation of PA28-capped 20S and PA700-20S proteasome complexes, we speculate that in addition to the ip also the function of the sp 20S core particle might be influenced by association with PA28 *in vivo*. We demonstrated that infected heart tissue is characterized by an increased abundance particularly of PA28β, which—as indicated by our infection experiments conducted in murine cardiomyocytes and HeLa cells—might directly interfere with the coxsackieviral replication machinery, all resulting in suppressed viral genome number and CVB3 VP1 protein levels. Facilitated liberation of the CVB3 epitope P3D_2170-2177_ within the context of epitope-harboring peptide processing by PA28-capped 20S proteasomes was also supportive for a biological function of PA28α/β during viral myocarditis. Nevertheless, *in vitro* findings on PA28α/β function were not corroborated by *in vivo* infection studies that investigated CVB3 myocarditis in constitutive PA28α/β^-/-^ mice. Other than reported for cytokine-inducible immunoproteasome formation [29, 31], PA28α/β is most likely not a prerequisite for coping with CVB3 myocarditis in C57BL/6 mice.

Induction of PA28α/β by IFN-γ has been observed in both immune and non-immune cells [14, 18]. Accordingly, increased abundance of PA28β in heart tissue obtained from CVB3-infected mice might be due to up-regulated IFN-γ-signaling active at this point in time of infection [5]. Presumably, such an increase in PA28 subunit abundance during inflammation might also involve a direct stimulation of PA28 α and β expression by signaling events mediated by pathogen-associated molecular patterns (PAMPs). Although such alternative pathways might be active during the initial phase of viral myocarditis, our observation that low-dose infection of cells with CVB3 leads to reduced PA28α/β mRNA and protein levels argues against a significant effect of CVB3-derived PAMPs in infected cells. Moreover, particularly the enteroviral protease 2A results in shut-off of Cap-dependent translation due to breakdown of the eukaryotic translation initiation factor eIF4G [53, 54]. Such mechanisms might contribute to reduced PA28α/β protein levels in CVB3-infected cells. Thus, IFN-mediated induction of PA28β expression most likely in non-infected cardiomyocytes contributes to increased PA28β abundance as found in infected heart tissue. There is also the possibility that increased PA28β abundance might be attributed to the infiltration of immune cells into infected heart tissue.

CVB3 infection of cardiomyocytes induces an increased formation of reactive oxygen species [unpublished observation] [55] known to be resulting in oxidant-damage of cellular proteins. The UPS plays an indispensable role for intracellular protein quality control through degradation of oxidized proteins that are potentially toxic [56]. This cellular process is of particular importance for pathophysiological conditions and might also be relevant during viral myocarditis [57]. Thus, we investigated TLR-3-activated BMM as a cell culture model for mild oxidative stress in immune cells to interfere with cellular homeostasis. Ablation of PA28α/β coincided with the detection of increased amounts of oxidized and poly-ubiquitinated proteins, thereby indicating for a putative function also of PA28-20S-PA700 particles for coping with disturbances in redox and protein metabolism. While our finding of decreased proteasome activity in naive splenocytes obtained from PA28α/β^-/-^ mice as evidenced by activity based probe profiling supports increased impairment of proteostasis, it is somewhat controversial to previous reports by the Goldberg and Cascio laboratories demonstrating that binding of PA28α/β to 20S or 20S-PA700 particles does not enhance the rate of protein degradation by the proteasome [21, 23]. Activity based probe profiling reflects the general accessibility of a compound to the catalytic active sites of the proteasome [34]. Both the Goldberg and Cascio laboratories used diverse biochemical approaches for investigating the function of PA28-proteasome complexes and such alternative experimental approaches might be attributed for the different findings on PA28 function *in vitro*. Other than previously reported for ip proteolysis in comparison to sp proteolysis in IFN-activated cells [58], increased levels of poly-ubiquitinated proteins found in TLR-3-activated BMM from PA28α/β^-/-^ mice had no effect on cellular metabolism and viability. Correspondingly, in CVB3-infected PA28α/β^-/-^ mice we found no biological phenotype that would be indicative for a putative failure of efficient protein turnover in these mice.

It is well established that UPS proteolysis can modulate the life cycle of intracellular pathogens [43, 44]. The biological significance particularly of the PA28α/β complex, however, remains to be defined [8]. In the present study, we found that PA28α/β has the capability to influence the abundance of intracellular viral RNA and protein during CVB3 infection, all being indicative for a putative role of this complex during viral replication. The elucidation of the underlying mechanisms is beyond the scope of this study. In contrast to divergent abundance of intracellular viral genome number and protein amounts in cells with altered PA28α/β expression, the amount of released viral particles was not affected by PA28α/β in cell culture infection models. Likewise, we found that ablation of PA28α/β in mice did not influence viral titers in heart tissue. We conclude that PA28α/β-proteasome particles might be capable to indeed modulate the coxsackieviral replication cycle. Nevertheless, the abundance of viral proteins during CVB3 infection seems to successfully counteract any putative protective cellular function of PA28α/β.

Another route how PA28α/β might affect viral infection is its proposed link to MHC class I antigen presentation [7–10]. We observed facilitated proteasomal generation of CVB3 P3D_2170-2177_ in the presence of PA28. Increased epitope liberation by PA28α/β is associated with enhanced substrate turnover, thus arguing against a specific alteration of cleavage site usage by PA28α/β-associated proteasome particles. The biological impact of this modulated antigen processing was investigated by infecting PA28α/β^-/-^ mice and their respective littermates. Our observation that ablation of PA28α/β had no impact on viral myocarditis and viral load indicates that facilitated antigen processing by PA28α/β-regulated proteasome particles has no profound biological impact during CVB3 infection. Interestingly, such discrepancies between *in vitro* and *in vivo* findings have also been reported earlier for the capacity of the ip regarding MHC class I antigen presentation during CVB3 infection [5, 29]. In contrast to unaltered CD8^+^ T cell responses found in mice with impaired ip function during CVB3 infection [29], the ip is well-known to efficiently facilitate antigen presentation in other infection models [3–6]. Taken into account that CVB3 elicits weak, presumably immuno-subdominant CD8^+^ T cell responses with unknown *in vivo* significance [5, 45, 46, 59], the mouse model of CVB3 infection might not be optimally suited for concluding on a general biological role both of PA28α/β and the ip for MHC class I antigen presentation.

According to *in vitro* data, the PA700-20S ip degrades unfolded proteins at a 10-fold higher rate than 20S ip and PA28-20S-PA28 ip complexes [21]. Therefore, PA700-20S ip represents presumably the most potent form of an immunoproteasome in terms of generating potentially immunogenic peptides [22]. Thus, differences in cleavage efficiency are attributed to changes in substrate gating [20] due to the association of PA700 with 20S particles. There is also substantial experimental evidence indicating accelerated protein turnover mediated by *de novo* formation of PA700-20S ip compared to their PA700-20S sp counterparts [47, 58]. The underlying molecular mechanisms for such effects are still poorly understood. The biological impact of altered peptide hydrolysis as a consequence of replacing the catalytic active sites in the sp with respective immunosubunits of the ip was investigated in LMP7^-/-^ mice [29, 31, 58], and thereby the influence of PA28α/β has not been addressed. We demonstrate in this study that PA28α/β associates both with 20S sp and 20S ip as well as with PA700-20S sp and PA700-20S ip particles, respectively. Together with the differential biological impact found during CVB3 myocarditis in LMP7^-/-^ mice [29, 31], we conclude that PA700 binding to 20S proteasomes [20] as well as formation of ip are biologically relevant ways to alter proteasome function. PA28α/β, however, is able to modulate total proteostasis in cells but is most likely a redundant component of the UPS during the pathogenesis of CVB3-induced acute myocarditis in C57BL/6 mice. Mice on this genetic background are resistant to the development of severe acute myocarditis [31] and this resistance is tightly connected to an efficient type I IFN response [36, 60] with a minor role of adaptive cellular immunity [57]. Thus, there is also the opportunity that mice on another genetic background with higher susceptibility to CVB3 infection might reveal an alternative *in vivo* function of PA28α/β during viral myocarditis that could possibly also reflect the *in vitro* findings of this study.
